# Complete resection of a giant intrapericardial cardiac synovial sarcoma

**DOI:** 10.1186/s13019-024-02725-8

**Published:** 2024-04-18

**Authors:** Binyue Wang, Ligang Liu

**Affiliations:** Department of Cardiovascular Surgery, Tongji Hospital affiliated to Tongji Medical College, Huazhong University of Science & Technology, No. 1095, Jiefang Avenue, Wuhan, Hubei Province 430030 China

**Keywords:** Cardiac tumor, Synovial sarcoma, Tumor resection, Case report

## Abstract

Synovial sarcoma of the heart is a rare tumor. Herein we would like to report a case of giant intrapericardial cardiac synovial sarcoma that originated from the right ventricle and grew outward near the diaphragm. After making adequate preoperative preparation, we performed the surgery as quickly as possible and resected the tumor completely. Based on the identification of the translocation on chromosome 18 rearrangement, the tumor can be diagnosed as a primary cardiac synovial sarcoma. Through this study, we aim to afford more information about cardiac synovial sarcomas as well as a reference for similar cases.

## Background

Synovial sarcoma (SS) is malignant and is categorized as a kind of soft tissue sarcoma. It often presents on the limbs [1], especially in the anatomy of the lower limbs near the knee-joint [2]. However, cardiac synovial sarcoma is rare. Herein, we present a case of a patient with a primary cardiac synovial sarcoma (PCSS).

## Case presentation

**Fig. 1:**
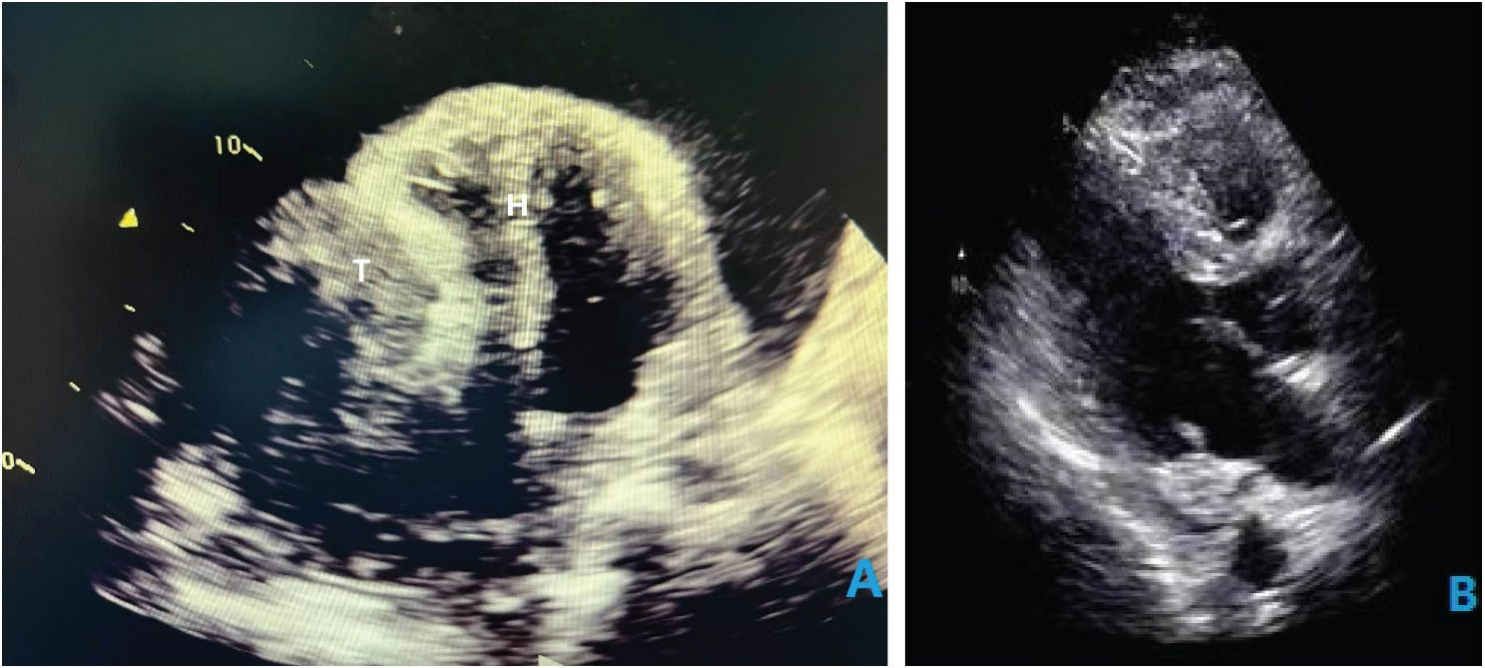
(**A**) Echocardiography showing intrapericardial space-occupying lesions. (**B**) Post-operative echocardiography. T: tumor; H: heart

**Fig. 2:**
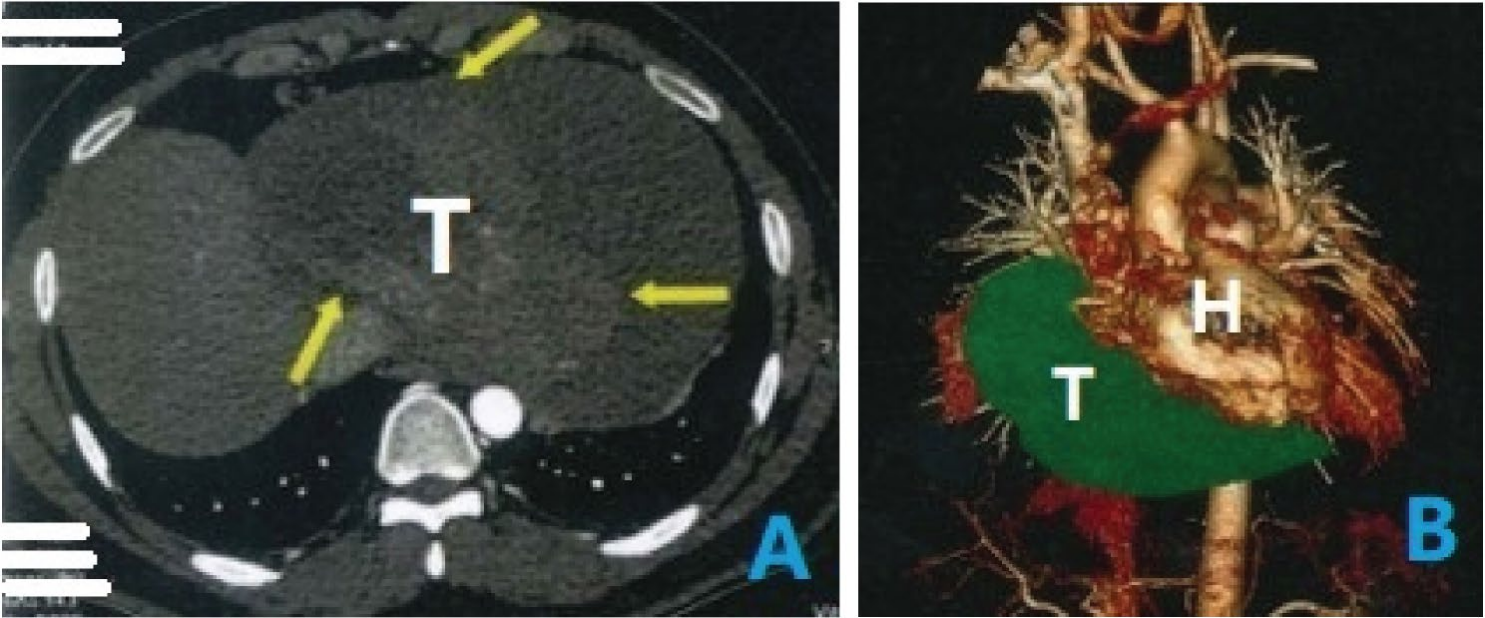
Plain computed tomography scan (**A**) and three-dimensional computed tomography scan (**B**) show a giant triangular pyramid mass adjacent to the ventricular diaphragmatic side of the heart. T: tumor; H: heart

A 35-year-old male patient visited our hospital, complaining of abdominal distention and edema of lower extremities for 1 month. Vital signs recorded included a blood pressure of 90/65mmHg and a heart rate of 110 beats per minute. Based on precordial auscultation, weak and distant heart sounds could be heard on the left chest wall. Upon physical examination, distention of jugular veins and compression edema of the lower extremities showed signs of pericardial tamponade. Upon experimental examination, the blood test results were abnormal, with elevated leukocyte count and reduced erythrocyte count. The electrocardiography showed nodal tachycardia. Transthoracic echocardiography indicated the presence of a giant mass, about 15 cm in size within the pericardium, located between the heart and the diaphragm, and the ventricles and atria were at normal size. The patient’s left ventricular ejection fraction was 63%. The echocardiography also showed massive pericardial effusion and the right heart was severely compressed (Fig. 1A). To relieve the pericardial tamponade, emergency pericardiocentesis was carried out and bloody pericardial effusion was revealed. After about 1000 ml fluid was removed, the patient felt much more comfortable with improved blood pressure, and decreased heart rate. So further examination could be carried out. To investigate more about the mass, we used a three-dimensional cardiac computed tomography (CT) scan and could conclude the presence of a distinct giant mass, about 15cmX20cmX10cm in size, triangular pyramid in shape, partly substantial and partly cystic, adjacent to the ventricular diaphragmatic side of the heart (Fig. 2).

**Fig. 3:**
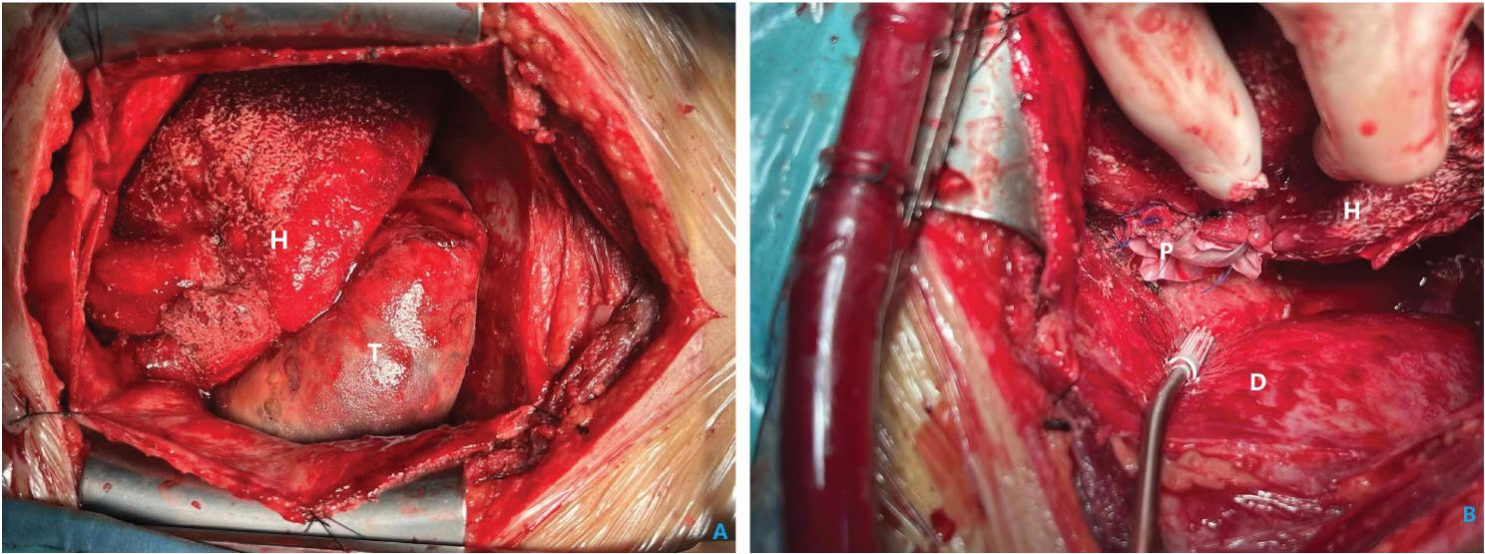
(**A**) During the operation, the tumor was observed between the heart and the diaphragm. (**B**) A bovine pericardial patch was used to repair the defect of the right ventricular wall after cutting the pedicle of the mass. H: heart; D: diaphragm; P: patch

Before undergoing a routine median sternotomy, the patient was well-prepared for surgery. After opening the pericardium and drainage of the bloody pericardial effusion, a giant triangular pyramidal mass, nearly the same size as the heart, was exposed. It was located between the heart and the diaphragm without any connection with the pericardium, but attached to the diaphragmatic side of the right ventricle (Fig. 3A). The pedicle was distant from the right coronary artery and the posterior descending artery. The size of the pedicle was about 4 cm in diameter. The mass was smooth, color-mixed, partly soft, and partly hard, and had a congestive hairy core.

**Fig. 4:**
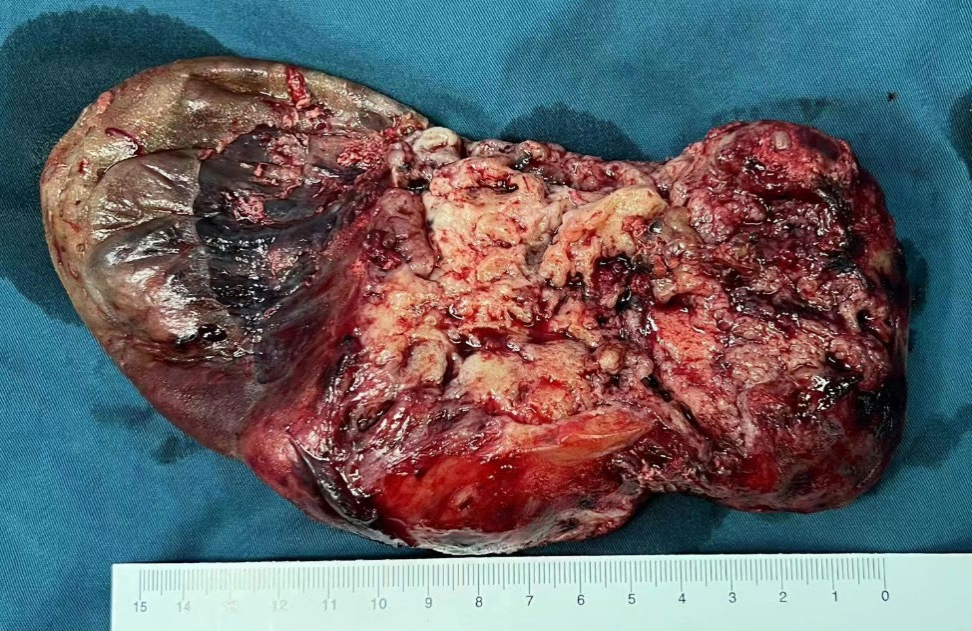
Surgical pathology. The mass is about 20cmX15cmX10cm in size

**Fig. 5:**
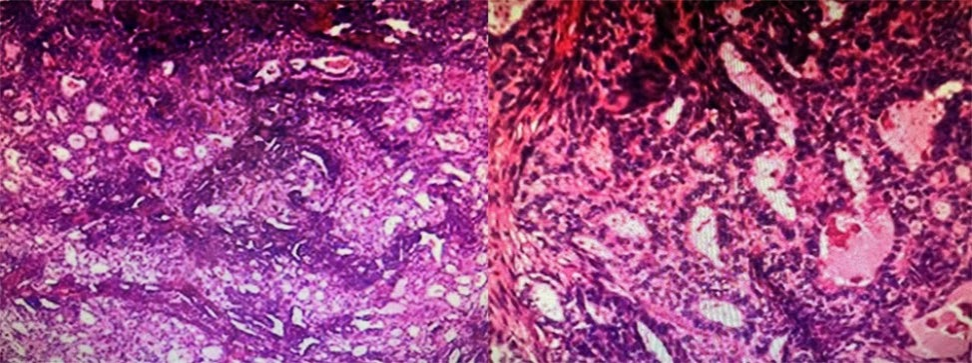
Pathology findings. Hematoxylin and eosin stain at intermediate power (100×) showing a biphasic synovial sarcoma morphology

**Fig. 6:**
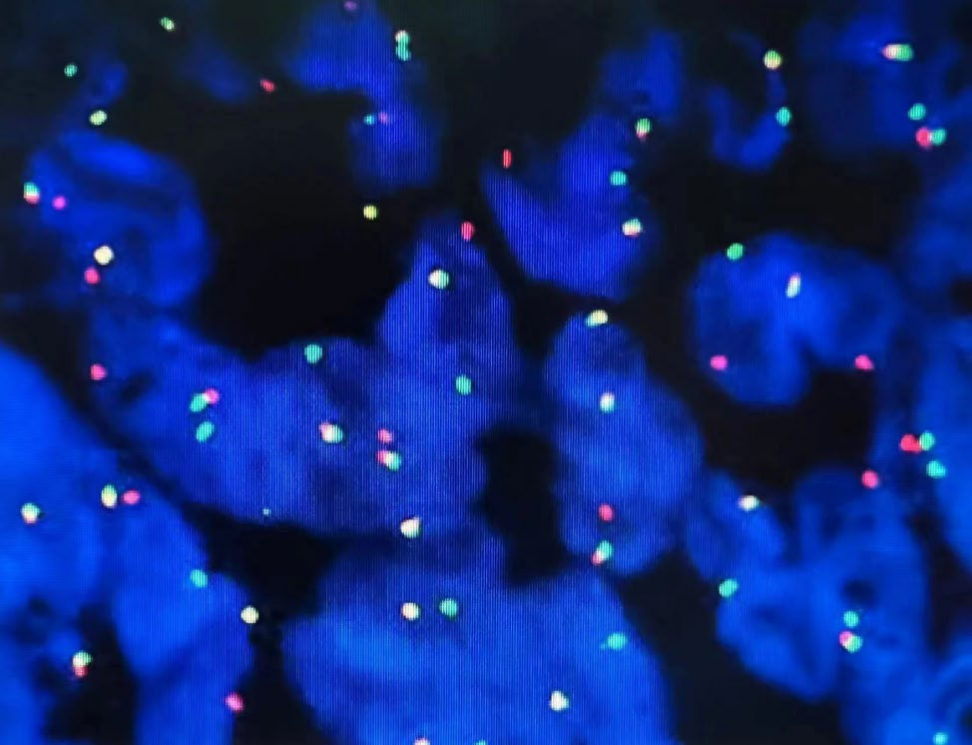
Fluorescence in situ hybridization showing rearrangement of the SS18 gene (split red and green signal)

To effectively control the possible perioperative hemorrhage and maintain hemodynamic stability, cardiopulmonary bypass was adopted. The superior and inferior vena cava were cannulated and the ascending aorta was chosen for traditional arterial cannulation. Surgical manipulation was performed on beating heart with normal body temperature, with the assistance of cardiopulmonary bypass. The pedicle of the mass was excised, and the relation between the mass and the cavity of the right ventricle was revealed. After extended excision of the ventricular wall adjacent to the pedicle, the mass was successfully removed en bloc. After that we plugged the defect with fingers to reduce bleeding and used an attractor to increase the clarity of the operative field. A bovine pericardial patch was then used to repair the defect of the right ventricular wall (Fig. 3B). In gross appearance, the mass was about 20cmX15cmX10cm in size, well encapsulated, partly cystic, and partly substantial, and appeared color-mixed (Fig. 4). On histopathological examination, it revealed the presence of tumor cells which were composed of spindle cells, with characteristics of being fasciculate, moderately heterotypic, frequently mitotic, and necrotic (Fig. 5). A strong positive response to cytokeratin AE1/AE3, TLE-1, and BCL-2 by the cells was detected through immunohistochemistry method, and the cells also had incomplete proactiveness to epithelial membrane antigen, WT-1, and ERG. An index of proliferation (immunostaining with Ki67) of about 15% was measured. The fluorescence in-situ hybridization test confirmed the diagnosis of a primary cardiac synovial sarcoma with a positive reaction to the t (X; 18) (p11.2; q11.2) genetic change (Fig. 6).

Postoperative course of this patient was uneventful. On the second day after surgery, he maintained a stable hemodynamics without right ventricular dysfunction, and the tracheal intubation was successfully removed. In-hospital post-operative echocardiography showed no obvious cardiac occupation but a small pericardial effusion of no big significance. The left ventricular ejection fraction was 64% (Fig. 1B). The patient was successfully discharged 10 days post-surgery. According to our follow-up, the patient declined any radiotherapy or chemotherapy for 6 months after discharge. He was diagnosed with a cardiac tumor again by echocardiography, and he received a second resection of cardiac tumor in another hospital, after which, he underwent 6 courses of chemotherapy. However, a more recent echocardiography still showed a tumor which was less than 1 cm in size in his heart. The good news is that the patient has survived more than 1 year and feels well.

## Discussion

SS can be dated from original mesenchymal cells. Based on the different composition of epithelial cells and spindle cells, SS can be categorized into 3 broad sections: poorly differentiated, single-phase, or biphasic. With mixed components of epithelial and spindle-cell, the tumor in our case was diagnosed as a biphasic cardiac SS [3]. The t (X; 18) (p11.2; q11.2) genetic change which leads to a merging of the SSX1 or SSX2 gene and the SS18 gene can be a special factor of SS because it has been observed in most cases of SS at present [4].

Cardiac masses are rare diseases and only 10–15% of them are malignant, with angiosarcoma being the most common malignant cardiac tumors [5]. These tumors are usually asymptomatic until they are big enough to cause symptoms [6]. PCSS is an extremely rare malignant entity that could involve the heart’s atrium and ventricles or the pericardium. For intracardiac cases, the right heart accounts for 71% of them and tends to arise mostly from the atrium [7]. And as of 2018, only 36 cases of pericardial synovial sarcoma have been reported in the literature [8]. The tumor discussed in our case, originated from the right ventricle and grew outward toward the diaphragm, which made it even more rare.

PCSS is predominant in male and has a high incidence in their 30s [9]. The clinical presentations of PCSS are nonspecific: dyspnea; heart failure; and pericardial effusion which could lead to cardiac tamponade. Compared with benign heart tumors, PCSS is more likely to present gastrointestinal and systemic symptoms. It can also readily result in hydropericardium and tends to grow bigger [10]. This patient exactly showed abdominal distention and massive bloody pericardial effusion. Chest x-rays images usually can only observe secondary signs of PCSS like cardiomegaly and pleural effusion. In the cases reported in previous studies, echocardiography was the first-line tool to use for diagnosis. Transthoracic bi-dimensional echocardiography has multiple benefits. It is non-invasive, low-cost, and can be performed at the bedside [11]. Also, transesophageal echocardiography can help to assess the tumor more clearly [12], but it is important to keep in mind that transesophageal echocardiography is an invasive procedure. CT is useful for evaluating calcification, and adjacent thoracic structure and checking for obstruction of coronary arteries [13]. Cardiovascular magnetic resonance imaging (cMRI) is more precise than echocardiography in detecting the form and actions of the heart [11]. cMRI can also evaluate the right heart better while echocardiography often neglects that [14]. Besides, cMRI and CT may be able to guide the surgical plan. Coronary angiography or cardiac CT angiography would be useful to delineate the anatomical correlation between coronary arteries and the location of the tumor. If the radiological images show the tumor has invaded coronary arteries, it would be difficult to excise the tumor completely. The final choice of image test depends on the experience of the surgeon and the facility of the hospital. Giving the patient’s symptom of pericardial tamponade, we initially only performed transthoracic bi-dimensional echocardiography. After the pericardiocentesis had relieved the pericardial tamponade, we performed a three-dimensional CT scan to get a clear anatomical relationship between the heart, great vessels, and the tumor, which guided the formulation of surgical plan.

At present, there are no clear protocols and guidelines to treat PCSS given its rarity. Surgical resection is still the most common treatment to serve for biopsy as well as to relieve symptoms in past cases. Complete surgical resection may be related to improved survival, but is usually tough, especially when the cardiac atria and ventricles are extensively involved. Before the operation, a good assessment of the relationship between the tumor and the near structures is necessary, because cardiac involvement may need cardiopulmonary bypass to completely resect the tumor [15]. The choice of operation is also important, median sternotomy has a high risk of postoperative infections and mediastinitis if radiotherapy is necessary [16]. Sometimes minimally invasive cardiac surgery can be an alternative. Adjunctive radiotherapy and chemotherapy have been lined to better survival, and SS seems to be more chemosensitive than most soft tissue sarcomas. Generally, a combination of ifosfamide and doxorubicin is the most commonly used chemotherapy regimen [9]. Targeted therapy is also being considered, Pazopanib is proven to be the only one to treat SS [17]. Heart transplantation is controversial, but some cases reported successful total artificial heart transplantation [18]. Before the patient underwent surgery, we made adequate preoperative preparation. During the operation, after exploring the extent of the tumor, we resected the mass completely. With the help of fluorescence in-situ hybridization testing, we finally diagnosed the tumor as a PCSS.

Most patients with SS had a dismal prognosis and died soon after being diagnosed [19]. Three independent factors (a young patient’s age, a complete resection, and application of adjuvant chemoradiotherapy) showed by an analysis can be associated with improved survival [7]. There is an urgent need for more effective ways to treat SS.

## Conclusion

We report a rare case where the tumor was completely resected surgically and the patient has survived more than 1 year. We want to provide some data to promote research on the treatment of PCSS.

## Data Availability

Data sharing is not applicable to this article as no datasets were generated or analysed during the current study.

## Abbreviations

SS: Synovial sarcoma
PCSS: Primary cardiac synovial sarcoma
CT: Computed tomography
cMRI: Cardiovascular magnetic resonance imaging
